# Supplementary figures and images for: The effects of allogenic stem cells in a murine model of hind limb diabetic ischemic tissue
Source: PeerJ. 2017 Aug 21;5:e3664. doi: 10.7717/peerj.3664 (PMC5572534; doi:10.7717/peerj.3664)

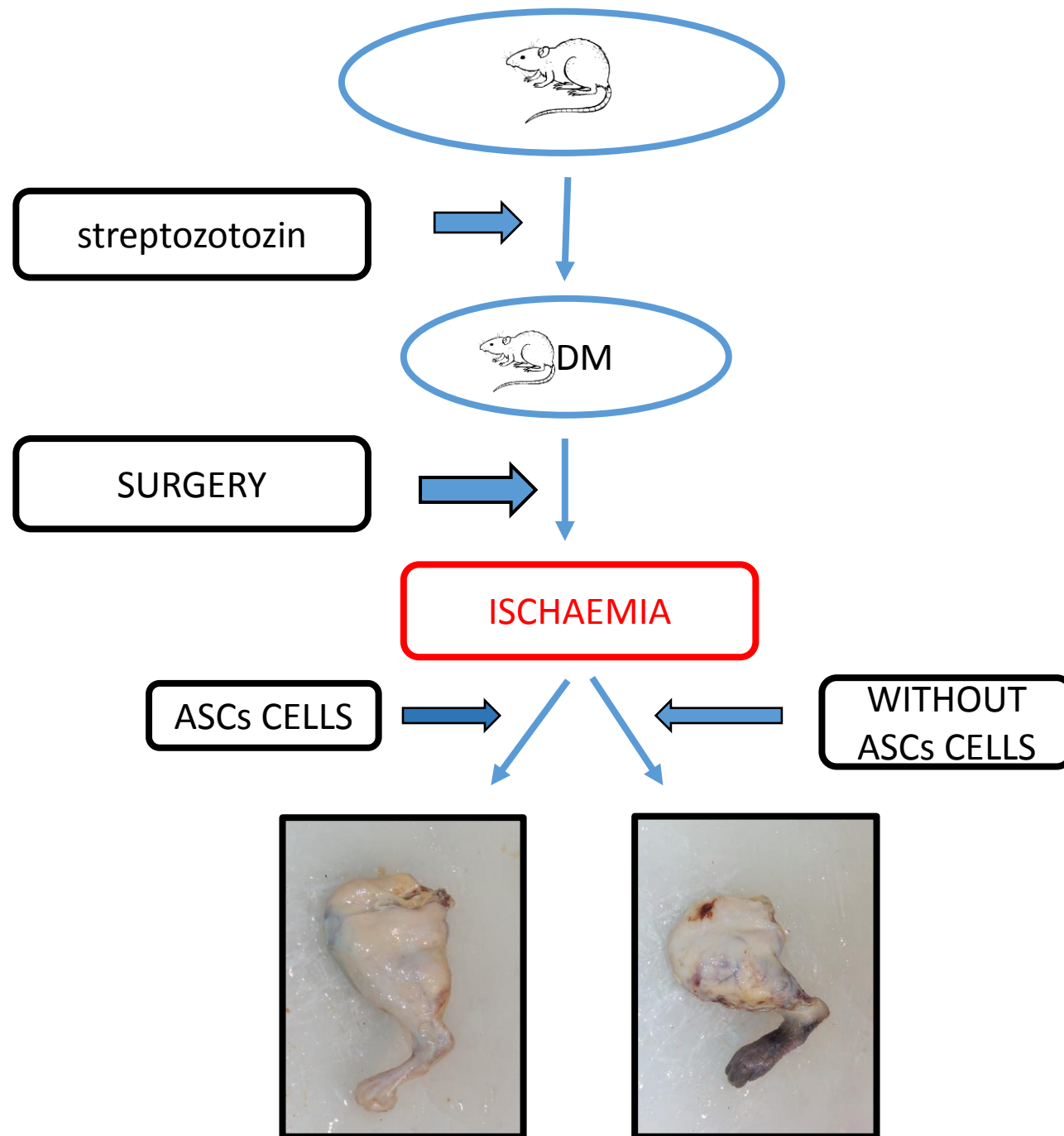

Supplement: Supplemental Information 1 [file peerj-05-3664-s001.pdf]
