# Supplementary material for: The effects of allogenic stem cells in a murine model of hind limb diabetic ischemic tissue
Source: PeerJ. 2017 Aug 21;5:e3664. doi: 10.7717/peerj.3664 (PMC5572534; doi:10.7717/peerj.3664)
Supplement: Supplemental Information 2 [file peerj-05-3664-s003.pdf]

Flow cytometry

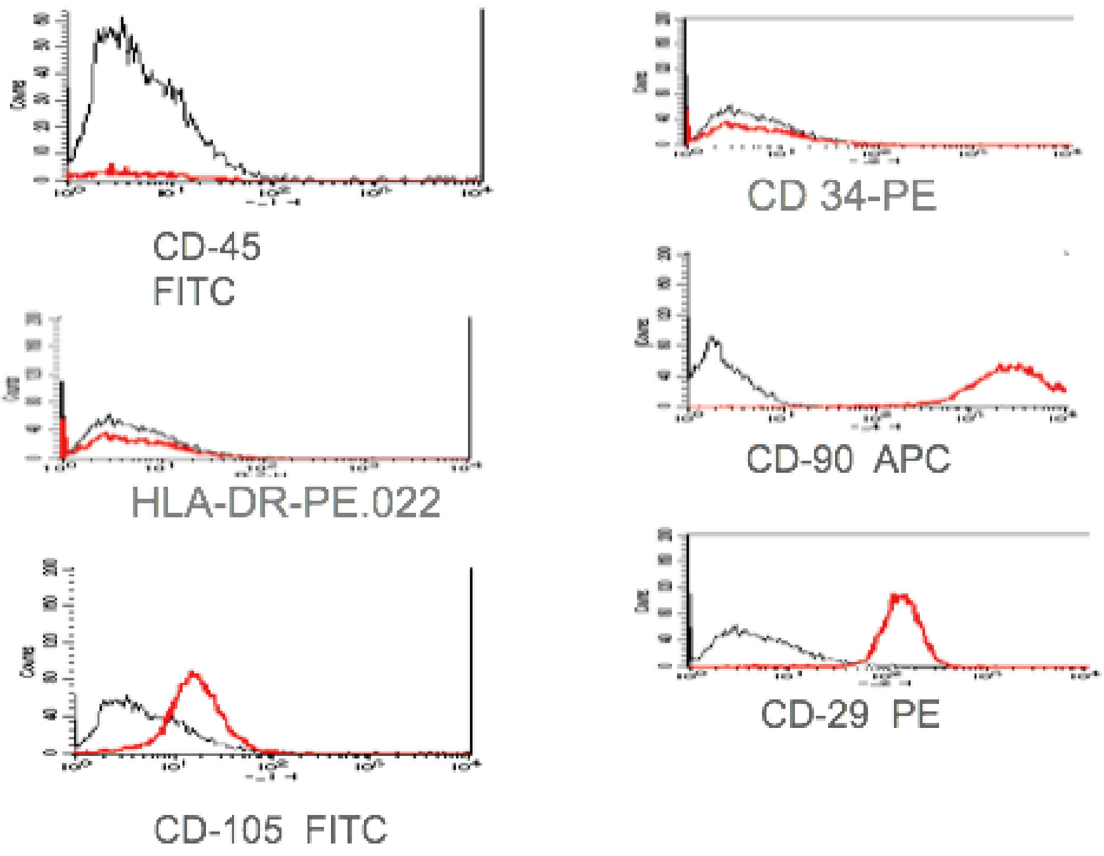

Legend: Once the cells were thawed and before being used in the treatments, we selected 200,000 cells and performed a study of 3 positive membrane markers (CD90, CD105 and CD29) and 3 negative membrane markers (CD34, HLA-DR and CD45), according to the IFATS criteria

Adipogenic differentiation

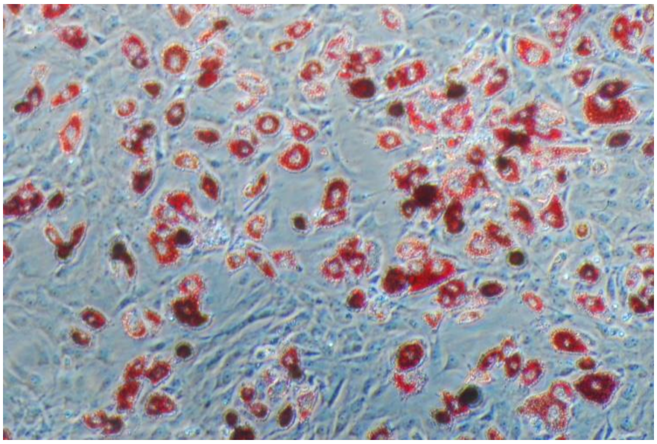

Osteogenic differentiation

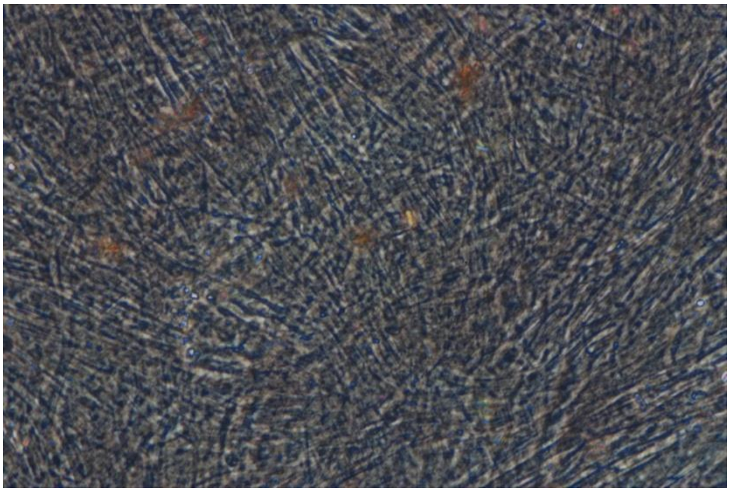

Legend: Adipogenic differentiation: lipid accumulation is visualized by staining with Oil Red O
Osteogenic differentiation: calcium-rich deposits secreted by osteocytes into the extracellular matrix were visualized by staining Alizarin Red S
